# Crosslinked Sulfonated Poly(vinyl alcohol)/Graphene Oxide Electrospun Nanofibers as Polyelectrolytes

**DOI:** 10.3390/nano9030397

**Published:** 2019-03-08

**Authors:** Oscar Gil-Castell, Diana Galindo-Alfaro, Soraya Sánchez-Ballester, Roberto Teruel-Juanes, José David Badia, Amparo Ribes-Greus

**Affiliations:** 1 Instituto de Tecnología de Materiales (ITM), Universitat Politècnica de València (UPV), Camino de Vera s/n, 46022 Valencia, Spain; ogilcastell@doctor.upv.es (O.G.-C.); diana_motiya@hotmail.com (D.G.-A.); rotejua@upvnet.upv.es (R.T.-J.); jose.badia@uv.es (J.D.B.); 2 Department of Chemical Engineering, School of Engineering, Universitat de València, Av. de la Universitat s/n, 46100 Burjassot, Spain; 3 Packaging, Transport, & Logistics Research Institute (ITENE), C/Albert Einstein, 1, Parque Tecnológico, 46980 Paterna, Spain; soraya.sanchez@itene.com

**Keywords:** poly(vinyl alcohol) (PVA), graphene oxide (GO), crosslinked, nanofibers, polyelectrolyte, proton conductivity

## Abstract

Taking advantage of the high functionalization capacity of poly(vinyl alcohol) (PVA), bead-free homogeneous nanofibrous mats were produced. The addition of functional groups by means of grafting strategies such as the sulfonation and the addition of nanoparticles such as graphene oxide (GO) were considered to bring new features to PVA. Two series of sulfonated and nonsulfonated composite nanofibers, with different compositions of GO, were prepared by electrospinning. The use of sulfosuccinic acid (SSA) allowed crosslinked and functionalized mats with controlled size and morphology to be obtained. The functionalization of the main chain of the PVA and the determination of the optimum composition of GO were analyzed in terms of the nanofibrous morphology, the chemical structure, the thermal properties, and conductivity. The crosslinking and the sulfonation treatment decreased the average fiber diameter of the nanofibers, which were electrical insulators regardless of the composition. The addition of small amounts of GO contributed to the retention of humidity, which significantly increased the proton conductivity. Although the single sulfonation of the polymer matrix produced a decrease in the proton conductivity, the combination of the sulfonation, the crosslinking, and the addition of GO enhanced the proton conductivity. The proposed nanofibers can be considered as good candidates for being exploited as valuable components for ionic polyelectrolyte membranes.

## 1. Introduction

The development of nanofibrous polyelectrolyte structures is a topic of interest in different fields of application, such as the preparation of proton exchange membranes for fuel cells [1], electrolysis membranes [2], or sensors [3], among others [4].

Electrospinning is one of the main approaches to obtain functionalized nanofibrous mats, including homopolymers, blends, or composites [5,6,7]. Given the wide versatility of the electrospinning technique, allowing the preparation of nanofibers of controlled size and morphology, the possibility of the development of functionalized polyelectrolytes has been explored [8].

Many materials have been considered for the production of nanofibrous polyelectrolytes, such as polyvinylidenefluoride (PVDF) [9], polystyrene (PS) [10], polysulfone (PSU) [11], sulfonated poly(ether sulfone) (SPES) [1], sulfonated poly(ether ether ketone) (sPEEK) [12], poly(vinyl butyral) (PVB) [13], and sulfonated polyimide (PI) [14]. Among them, the use of poly(vinyl alcohol) (PVA) [15,16,17] has been considered as an economically feasible alternative with promising results in terms of cost, versatility, processability, and capability of functionalization [18].

Regarding its application, PVA requires functionalization to incorporate specific ionic domains to induce polyelectrolyte performance. Different strategies, such as copolymerization, polymer grafting, or combining with inorganic particles to create composite materials, have been proposed [19]. The sulfonation of PVA can be reached by means of the grafting of sulfonic domains at the hydroxyl side group of the polymer backbone [20]. In addition, the crosslinking strategy of the PVA with agents containing two or more reactive functional groups has been reported [21]. PVA has been crosslinked with glutaraldehyde (GA) [22,23,24,25], poly(acrylic acid) (PAA) [26,27], polystyrene(sulfonic acid co-maleic acid) (PSSA-MA) [28,29], chitosan (CS) [30,31], and glyoxal [32], among others [19,33]. One of the most interesting crosslinking agents of PVA is sulfusoccinic acid (SSA) [34,35,36], which brings sulfonic groups to the polymer structure that regulate water uptake ability, selectivity, and ionic transport capacity [37,38,39]. In addition, the incorporation of charges or reinforcements in the polymer matrix can provide an improvement in the barrier and selectivity effect to the diffusion of determined substances through the membrane [40]. These multicomponent systems can present synergic properties that optimize their performance [41,42].

Graphene oxide (GO) has received considerable interest because of its unique two-dimensional structure with excellent dimensional, chemical, thermal and mechanical stability, low permeability, low cost, surface functionality, and minimum thickness [43]. GO can be produced from graphite using several oxidation routes in a relatively easy way [43,44], and its strong hydrophilic properties provide good dispersion in water and improve interfacial adhesion in hydrophilic polymer matrices, such as PVA [45,46,47].

In this context, raw and sulfonated PVA combined with graphene oxide (GO) and crosslinked with sulfosuccinic acid (SSA) have been proposed in this work for developing functionalized nanofibers using the electrospinning technique [17,30,46,47,48,49]. The sulfonic group introduction both as a crosslinking agent and also grafted to the PVA backbone and the effect of the graphene oxide nanoparticles with a highly hydrophilic surface will be carefully assessed in terms of specific behavior for its use as polymer-based electrolytes.

## 2. Materials and Methods

### 2.1. Obtaining of Nanofibers

#### 2.1.1. Materials

The poly(vinyl alcohol) (PVA) (99% hydrolyzed, *M_w_* 130,000 g·mol^−1^) was supplied by Sigma Aldrich (Madrid, Spain). The sulfonation of poly(vinyl alcohol) (PVA) was performed with the use of 1,3-propanesultone 97%, purchased from Acros Organics (Thermo Fisher Scientific, Geel, Belgium). The sulfonation reaction was carried out in two stages that have been described elsewhere [50,51]. Graphene oxide (GO) was obtained by the modified Hummers method [44], following the procedure described before [39].

#### 2.1.2. Solution Preparation

A main aqueous solution of 8%wt of PVA in 50 mL of deionized water was prepared and stirred at 90 °C for 6 h in a reflux system. After that, a 0.25%wt_PVA_ of a non-ionic surfactant Triton^®^ X-100 was added to reduce the surface tension of the solution [15,52]. To achieve crosslinking and to increase the proton conductivity of the developed nanofibers, a 30%wt_PVA_ of sulfosuccinic acid (SSA) was added to the dissolution and homogenized under stirring for 24 h [19,37].

Simultaneously, the GO solutions were prepared with a concentration of 0.25, 0.50, 0.75, and 1.00%wt_PVA_ in 10 mL of deionized water to achieve a good dispersion. These GO solutions were sonicated for 30 min in a ATM40-2LCD ultrasonic bath (ATU Ultrasonidos, Paterna, Spain). Subsequently, the GO solutions were added to the PVA dissolution and stirred for 30 min. Taking into account the addition of 10 mL of the GO solution to the mixture of PVA/Triton^®^/SSA, the concentration of the latter was adjusted to achieve the desired final concentrations. The same process was repeated for the sulfonated PVA (SPVA), with equal concentrations of Triton^®^, SSA, and GO, as defined for PVA.

#### 2.1.3. Electrospinning

**Figure 1:**
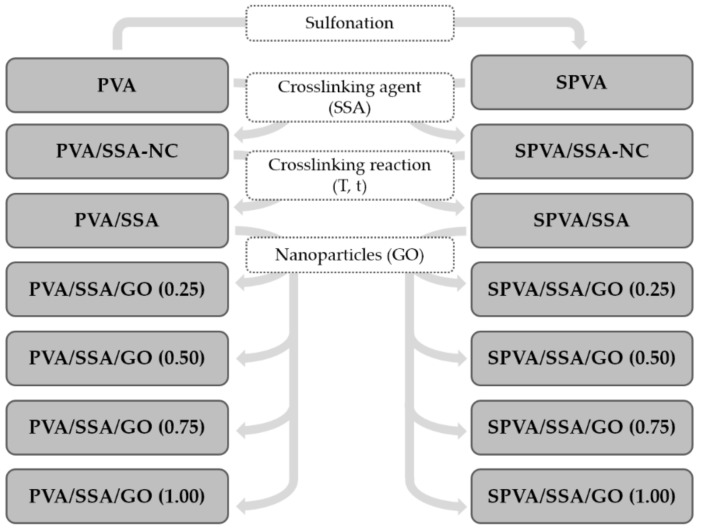
Fiber composition and studied effects on the physico-chemical properties: matrix sulfonation, addition of sulfosuccinic acid (SSA), crosslinking reaction, and addition of graphene oxide (GO) nanoparticles. (NC stands for non-crosslinked.)

The electrospinning process was carried out by means of a Fluidnatek LE-10 equipment (Bionicia, Paterna, Spain). The solutions cited in the previous section were taken and each filled a plastic syringe (5 mL) with a diameter of 11.99 mm (Becton Dickinson Iberia, Madrid, Spain). The distance from the tip of the needle to the flat collector was set at 17 cm. The electrospinning was carried out with a variable feeding rate between 300 and 800 μL·h^−1^ and a voltage ranging from 16 to 18 kV. The time of electrospinning varied as a function of the flow rate, in order to obtain the same quantity of electrospun material in all the samples, and ranged between 2 and 3 h. A nanofibrous mat of 4 × 4 cm^2^ surface with a thickness of approximately 20 μm was obtained in all cases. Figure 1 summarizes the designation of the nanofibers obtained in the present study. Once obtained, they were saved in zip bags and stored in a desiccator at room temperature for subsequent steps.

#### 2.1.4. Nanofiber Crosslinking

The PVA/SSA and SPVA/SSA-based nanofibers were subjected to a crosslinking reaction to provide sulfonic functional groups and bring stability. For this purpose, the nanofibrous mats were placed between two sheets of Teflon^®^ reinforced with glass fiber and, in turn, introduced between two metallic discs to ensure the crosslinking reaction in a planar arrangement. The entire assembly was then subjected to 110 °C for 2 h in a UT-6060 convection oven (Heraeus, Hanau, Germany). Finally, the crosslinked nanofibers were stored in zip bags and placed into a desiccator at room temperature for further analyses.

### 2.2. Analytical Assessment

#### 2.2.1. Field-Emission Scanning Electron Microscopy (FE-SEM)

The field-emission scanning electron microscopy (FE-SEM) was performed by means of a Zeiss Ultra 55 microscope (Carl Zeiss, Jena, Germany). Nanofibers were mounted onto metallic sample holders and sputter coated with a K950 Sputter Coater device (Mitek, San Diego, CA, USA) under an inert atmosphere and vacuum conditions. Samples were platinum coated for 15 s. The micrographs were taken with a working distance of 7 mm and a voltage of 2 kV at different degrees of magnification. The diameter of the nanofibers was obtained from the average of 100 measurements by means of the Image J software. Three different electron microscopy images representative of different regions of the nanofibrous mat were analyzed.

#### 2.2.2. Fourier-Transformed Infrared Spectroscopy (FT-IR)

The Fourier-transformed infrared spectroscopy (FT-IR) was performed in a Nicolet 5700 spectrometer (Thermo Fisher Scientific, Waltham, MA, USA), equipped with a total attenuated reflectance (ATR) module. The FT-IR spectrum was taken between 4000 and 400 cm^−1^ and a resolution of 4 cm^−1^ along 64 scans. Five assays were performed from different points in the same sample and the mean spectra were taken as representative.

#### 2.2.3. Thermogravimetry (TGA)

The thermogravimetric assays (TGA) were performed by means of a TGA 851 analyzer (Mettler Toledo, Columbus, OH, USA). The samples (5–8 mg) were introduced into 70 μL alumina capsules and subjected to a dynamic assay, based on a heating segment from 25 °C to 800 °C with a heating rate of 10 °C·min^−1^. The tests were carried out under an oxidative atmosphere with an O_2_ feeding rate of 50 mL·min^−1^. The samples were studied in triplicates and the averages and deviations were taken as representative values.

#### 2.2.4. Differential Scanning Calorimetry (DSC)

The differential scanning calorimetry (DSC) was performed in a DSC 822^e^ setup (Mettler Toledo, Columbus, OH, USA) equipped with a refrigeration system. The samples (3–5 mg) were placed into 40 μL aluminum crucibles. The method of analysis consisted of different consecutive heating/cooling/heating segments between 50 °C and 250 °C with a heating/cooling rate of 10 °C·min^−1^. The assays were carried out under an inert atmosphere of N_2_ at a flow rate of 50 mL·min^−1^. The samples were studied in triplicates and the averages and deviations were taken as representative values.

#### 2.2.5. Electrical and Proton Conductometry

The proton conductivity (*σ_prot_*) was measured by means of a dielectric thermal analyzer (DETA) composed of an alpha mainframe frequency analyzer in conjunction with a Concept 40 active cell (Novocontrol Technologies, Montabaur, Germany). The response was measured in the frequency range from 10^−2^ to 10^7^ Hz at room temperature (25 °C). The sample electrode assembly (SEA) consisted of two stainless steel electrodes filled with the polymer. The diameter of the electrodes was 20 mm and the thickness was kept around 30 μm. Preliminarily, the polyelectrolytes were hydrated in ultra-pure water for 24 h. The *σ_prot_* (S·cm^−1^) was calculated according to Equation 1:

(1) $$\sigma_{prot}=\frac{l}{A\cdot R_{0}}$$

where *l* is the thickness of the conducting nanofibrous membranes in cm, *A* is the area of the electrode in contact with the sample in cm^2^, and *R_0_* is the proton impedance taken from the Bode plot at high frequencies in ohms (Ω) [53].

The electrical conductivity (*σ_elec_*) was measured at room temperature using the same equipment, at low frequencies, where the measured real part of the conductivity (*σ*’) reaches a plateau that is strictly correlated to the direct current (DC) conductivity (*σ_0_*).

#### 2.2.6. Immersion in Simulated Service Conditions

The characterization of the stability of the nanofibers under simulated service conditions was assessed by means of the immersion in ultra-pure water, which can be correlated to the performance of these materials for application as polyelectrolytes in fuel cells [54]. For this purpose, the samples with a surface of 4 × 4 cm^2^ were introduced in test tubes filled with water and placed at 60 °C in a thermostatic Selecta Unitronic Bath for 120 h. Afterwards, they were withdrawn from the tubes, the surface water was removed, and they were dried under vacuum at 30 °C for 48 h. The changes after immersion were characterized in terms of the variation of the nanofiber morphology by means of field-emission scanning electron microscopy (FE-SEM), as previously described.

## 3. Results and Discussion

In this study, the effects of the addition of SSA to the PVA matrix, the crosslinking reaction, the sulfonation of the PVA (SPVA), and the combination with GO nanoparticles on the electrospun nanofibers—summarized in Figure 1—were assessed in terms of its suitability for being used as polymer electrolytes. The discussion is given from different perspectives, including studies such as the surface morphology, the chemical composition, the thermal properties, the thermo-oxidative stability, the electrical and proton conductivity, and the stability when submitted to simulated service conditions.

### 3.1. Surface Morphology

**Figure 2:**
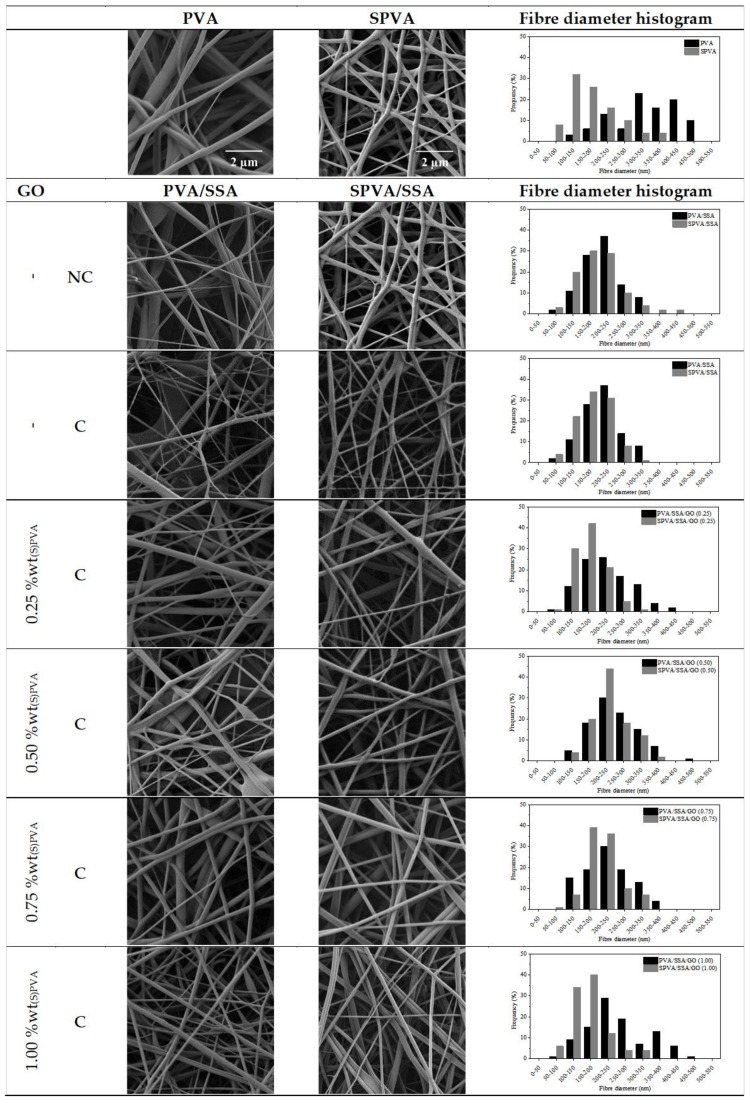
Surface morphology (10,000×) and fiber diameter histograms of the nanofibers of poly(vinyl alcohol) (PVA) and sulfonated PVA and their corresponding GO-based composites. (C stands for crosslinked and NC for non-crosslinked; 10,000×.)

The surface morphology of the obtained PVA/SSA/GO and SPVA/SSA/GO nanofibers was characterized by means of field-emission scanning electron microscopy (FE-SEM). The acquired electronic micrographs, along with the fiber diameter histograms are gathered in Figure 2.

In general, smooth bead-free electrospun nanofibers were obtained. The SPVA-based nanofibers showed histograms displaced towards lower values in comparison to those of PVA. The SPVA showed an average diameter around 200 nm while that of pure PVA nanofibers was around 350 nm. This effect can be correlated to the higher conductivity of the SPVA solution during electrospinning [50].

For the crosslinked PVA/SSA and SPVA/SSA nanofibers, the average fiber diameter decreased by 10%. Crosslinking may have reduced the free volume inside the fibers and, subsequently, resulted in a more compact structure due to the intra- and inter-molecular chemical interactions between the PVA and the SSA [55,56]. Additionally, the feasible release of remnant water molecules during the crosslinking reaction at 110 °C may have contributed to the reduction of the fiber diameter. The addition of the GO nanoparticles slightly modified the nanofiber diameter.

### 3.2. Chemical Composition

**Figure 3:**
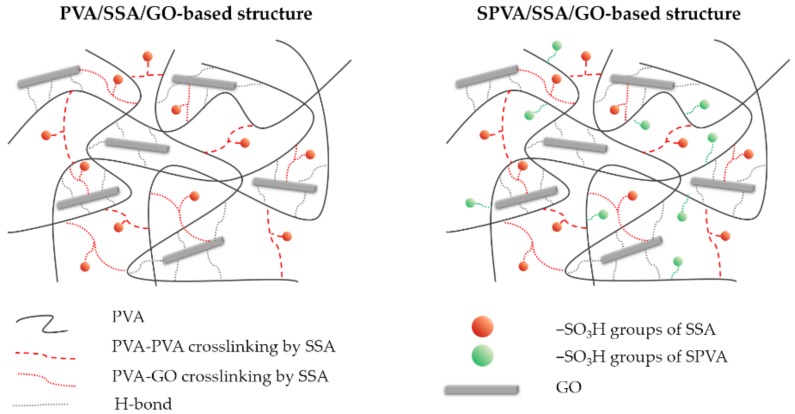
Proposed model structure of the PVA and sulfonated PVA (SPVA)-based crosslinked composite membranes.

**Figure 4:**
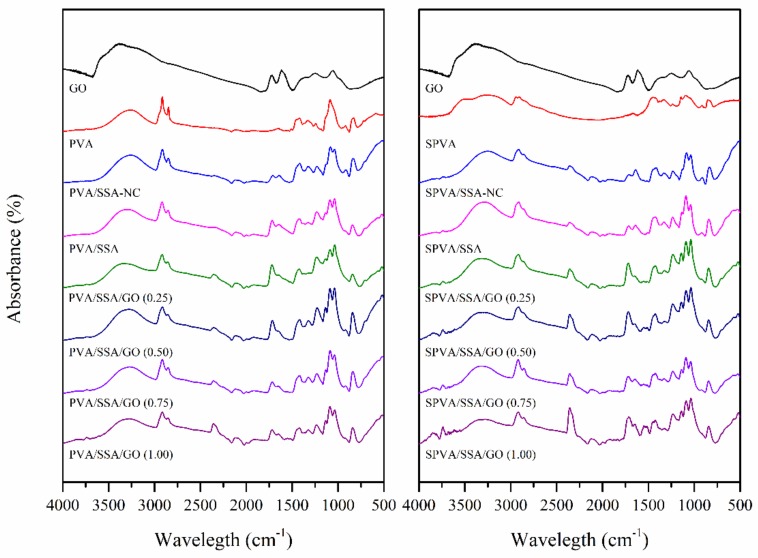
Infrared absorbance spectra of the GO and the nanofibers of PVA and sulfonated PVA and their corresponding GO-based composites. (NC stands for non-crosslinked.)

Figure 3 represents a proposed structural model of the PVA and SPVA-based crosslinked composite nanofibers. In order to validate this model, the chemical composition of the produced nanofibers as well as that of the GO, the PVA, and the SPVA was assessed by means of Fourier-transformed infrared (FT-IR). Figure 4 shows the obtained infrared spectra.

Both PVA and SPVA-based nanofibers, revealed a wide absorption band between 3000 and 3400 cm^−1^, associated with the stretching of the ‒OH groups of PVA. The peaks between 1660 and 1640 cm^−1^ are correlated to the ‒OH bending of the H_2_O molecules retained in the material, due to its high hydrophilicity. The peaks between 2800 and 3000 cm^−1^ and between 1300 and 1500 cm^−1^ corresponded to the stretching and deformation vibrations of the ‒CH_2_‒ groups of the PVA molecule, respectively [50]. The peak around 1080 cm^−1^ can be associated with the vibration stretching of the C‒O bonds of the PVA [57]. For the SPVA, an intense peak around 1040 cm^−1^ related to the presence of sulfonic groups (‒SO_3_H) was observed, confirming the sulfonation strategy for this material [21].

When the SSA was added to the nanofibers, an increase of the bands between 1780 and 1710 cm^−1^, associated with the carboxyl (‒COOH) and acetate (‒COO‒) groups, was observed. The stretching vibration of the O‒H bond of the carboxyl groups appeared between 2500 and 3000 cm^−1^. In addition, an increase in the peak intensity of the 1040 cm^−1^ signal associated with the stretching of the sulfonic groups (‒SO_3_H) was perceived [58]. After crosslinking, the intensity of the stretching vibration signal of the –OH group of the PVA molecules was reduced, given its reaction with the carboxyl group of the SSA molecules [50].

The GO spectrum was characterized by peaks at 3450, 1720, 1600, 1400, and 1100 cm^−1^, assigned to the –OH group stretching vibrations, stretching vibrations of C=O, skeletal vibrations of nonoxidized graphite domains and C=C bonds, and to deformation vibrations of the O‒H and C‒O bonds, respectively [45,59]. Given the low percentage of GO in the nanofibers, the representative peaks of the nanoparticles overlapped. This absence may be also correlated to a good dispersion of GO in the PVA matrix.

### 3.3. Thermal Properties

**Figure 5:**
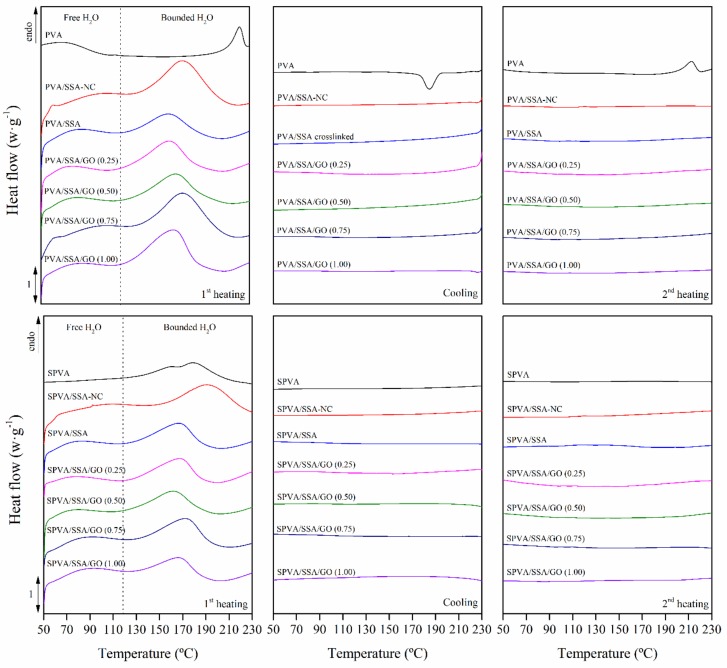
Calorimetric thermograms of the first heating (left), cooling (center), and second heating (right) scans of the nanofibers of PVA and sulfonated PVA and their corresponding GO-based composites. (NC stands for non-crosslinked.)

The thermal properties of the nanofibers were studied by means of differential scanning calorimetry (DSC), which brings valuable information about the microstructure and morphology of the polymer-based materials [60]. The thermograms obtained for the PVA, PVA/SSA, PVA/SSA/GO, and SPVA/SSA/GO nanofibers are shown in Figure 5. The effects of the addition of SSA, the crosslinking, the sulfonation of the PVA, and the addition of GO were studied.

The pure PVA nanofibers showed an endothermic peak between 50 and 100 °C, associated with the evaporation of free water, followed by an endothermic peak at 229 °C, ascribed to the melting of crystalline domains. In the cooling scan, a crystallization peak was shown with its maximum located at 191 °C. Finally, when the thermal story and water content were removed, a melting peak was observed with its maximum at 220 °C. These results confirmed the typical semicrystalline morphology of the pure PVA [36].

In contrast, the SPVA nanofibers revealed an amorphous morphology due the absence of melting and crystallization peaks. The hydrogen bonding interactions between the PVA molecules and the sulfonic groups may have prevented the PVA from crystallization [50]. Indeed, a solely endothermic peak between 150 °C and 200 °C was observed, which was associated with the release of bound water.

The crosslinked nanofibers also revealed an amorphous morphology, as reported for PVA with concentrations of SSA greater than 15% [34,36,61]. Free and bound water release processes were found. The free water is simply retained physically inside the nanofibers, while the bound water is chemically linked to the hydroxyl groups of the PVA and to the SSA molecules by hydrogen bonding. Both processes were displaced towards lower temperatures, as gathered in Table 1. The reaction between the hydroxyl groups of the PVA and the carboxyl groups of the SSA reduced the interaction capability with water molecules [34,61].

The PVA/SSA/GO and SPVA/SSA/GO nanofibers showed the absence of melting and crystallization peaks in the cooling and heating scans, and therefore an amorphous morphology is expected for these materials [34,36,61]. Only the characteristic endothermic peak of the free and bound water release processes was observed. The huge amount of hydroxyl groups in the GO nanoparticles surface brought a stronger interaction with water molecules and slightly increased the bound water release temperature.

### 3.4. Thermo-Oxidative Stability

**Figure 6:**
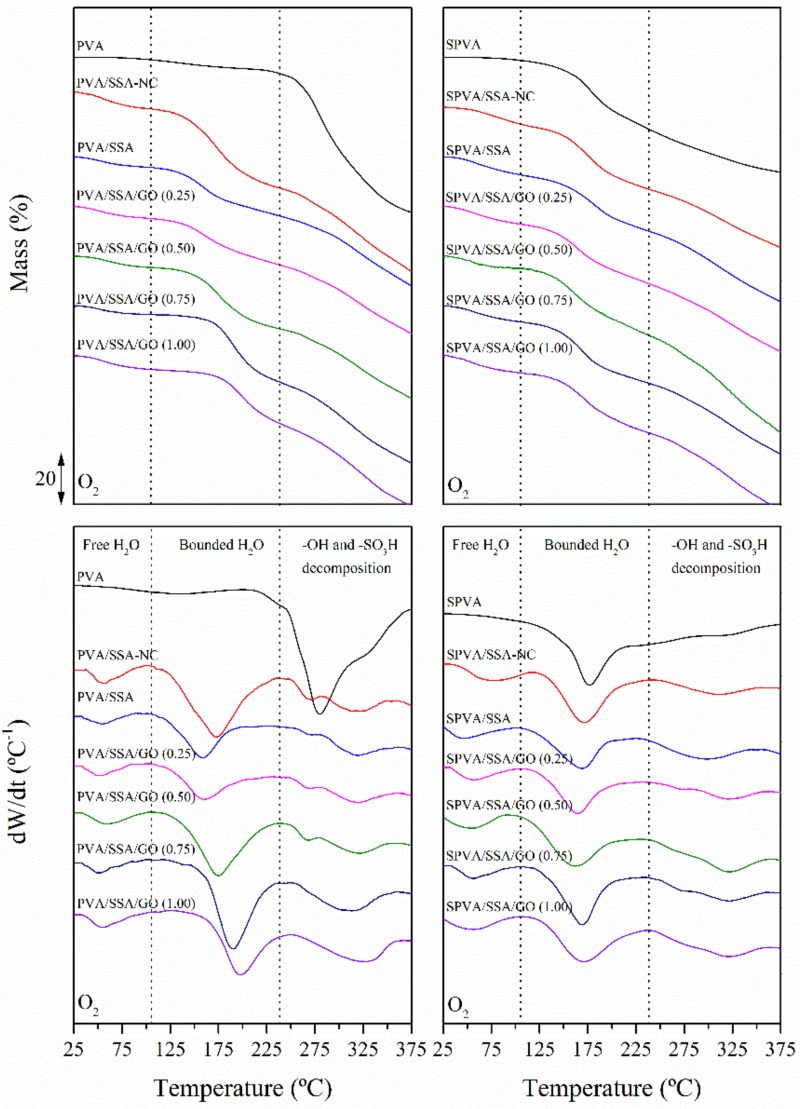
Thermogravimetric (TG) (up) and first derivative thermogravimetric (DTG) (down) curves of the nanofibers of PVA and SPVA and their corresponding GO-based composites. (NC stands for non-crosslinked.)

The thermo-oxidative stability was further assessed by means of thermogravimetry (TGA). The thermogravimetric curves (TG) along with the first derivative thermogravimetric curve (DTG) obtained under an oxidative atmosphere for all the developed nanofibers are plotted in Figure 6. The decomposition profiles were characterized in terms of the mass loss from the TG curves and the characteristic temperatures from the peaks of the DTG curves. These results agree with the literature [21,61] and are gathered in Table 2.

The neat PVA nanofibers underwent a first mass loss stage between 100 and 200 °C and comprised a gradual loss of free and bounded water, respectively. The second degradation stage, between 250 °C and 300 °C, was dominated by the cleavage reactions of the ‒OH groups of the PVA molecules. The thermogram of the SPVA nanofibers revealed an additional peak around 300 °C related to the decomposition of the grafted ‒SO_3_H groups.

The crosslinked nanofibers showed thermo-oxidation behavior in multiple stages. The first stage with a mass loss of around 10% was observed from 50 to 100 °C. In the second stage, between 100 and 230 °C, a mass loss of 20% took place, attributed to bounded water release. In this case, water molecules are assumed to be bonded to the polymer chains in the available hydroxyl, sulfonic, and carboxylic groups by hydrogen bonding [34]. The third region of mass loss from 230 to 350 °C was associated with the desulfonation process, the degradation of hydroxyl groups, and the breakage of the crosslinking ester bonds [61]. In general, the crosslinked nanofibers revealed a slightly higher mass loss during the desulfonation process, which supports the presumed greater amount of sulfonic groups in these materials [46]. Finally, from 350 °C onwards, the degradation of the polymeric backbone of the PVA took place.

Regarding the PVA/SSA/GO and SPVA/SSA/GO nanofibers, the mass loss of the water release stage remained almost constant. However, the bound water release temperature increased as a function of the GO content. The functional groups of the GO nanoparticles available to interact with water molecules may have formed stronger hydrogen bonds, which may have inhibited the release of water.

### 3.5. Electrical Conductivity

**Figure 7:**
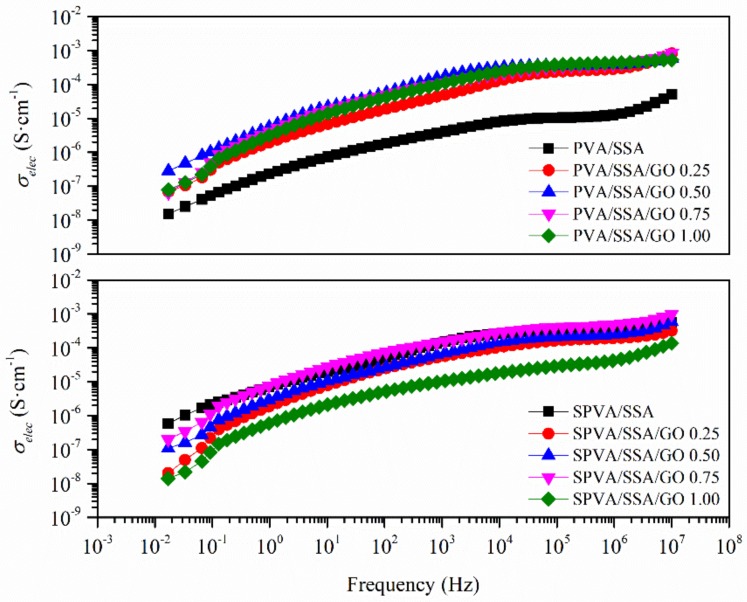
Electrical conductivity (*σ_elec_*) as a function of the frequency of the nanofibers of PVA and sulfonated PVA and their corresponding GO-based composites.

The electrical conductivity (*σ_elec_*), as obtained by means of dielectric thermal analysis (DETA), is plotted in Figure 7 as a function of the frequency. The *σ_elec_* was considered at low frequencies, where the measured real part of the conductivity (*σ*’) reached a plateau that was strictly correlated to the DC conductivity (*σ_0_*). All the developed nanofibers revealed an *σ_elec_* in the range between 10^−7^ and 10^−8^ S·cm^−1^. Therefore, they can be considered as good insulators that prevent the flow of electrons through them, especially relevant for some polyelectrolyte applications.

### 3.6. Proton Conductivity

**Figure 8:**
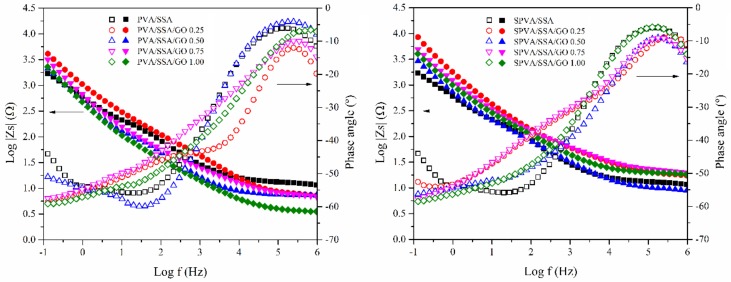
Bode diagrams for the PVA/SSA (left) and SPVA/SSA (right) based composite nanofibers.

There are two main proton conduction pathways in the PVA/SSA and SPVA/SSA-based polyelectrolytes [33]. On the one hand, the protons may interact through the jump (or Grotthuss) mechanism between the sulfonic groups, both from the SSA and from the sulfonation of PVA. On the other hand, they can use the vehicle mechanism through the hydroxyl and carboxyl groups of the PVA, the SSA, and the GO, which may interact with water molecules, boosting the proton conductivity (*σ_prot_*). Thus, the *σ_prot_* will strongly depend on the degree of hydration of the material. For this reason, the nanofibers were measured after being subjected to a hydration process through immersion for 24 h in ultra-pure water. Afterwards, the effects of the sulfonation of the PVA, the addition of the SSA, as well as the incorporation of the GO with respect to the proton conductivity were studied. Figure 8 represents Bode diagrams for the PVA/SSA/GO and SPVA/SSA/GO nanofibers, showing the real part of the impedance and the phase angle as a function of the frequency at 25 °C. The real impedance reached a constant value and the phase angle reached a maximum close to zero for high frequencies. The *σ_prot_* values, as calculated by means of Equation (1) are shown in Table 3.

The *σ_prot_* of the proposed PVA-based nanofibers must still be enhanced if compared to Nafion^®^-based nanofibers [9,62]. However, according to the literature, the obtained *σ_prot_* of the PVA/SSA/GO nanofibers may be comparable to that found for sulfonated poly(ether sulfone) (SPES) (9.3 × 10^−4^ S·cm^−1^) [1], poly(vinylidene fluoride) (PVDF)/phosphotungstic acid (PWA) (3.0 × 10^−4^ S·cm^−1^) [63], sulfonated poly(ether ether ketone) (SPEEK) (2.5 × 10^−3^ S·cm^−1^) [12], and sulfonated poly(arylene ether sulfone) (SPAES) (5 × 10^−4^ S·cm^−1^) [11], all of which were measured at room temperature.

The *σ_prot_* of the PVA/SSA/GO nanofibers progressively increased as a function of the GO content. In contrast, for SPVA/SSA/GO nanofibers, the *σ_prot_* depended on the proportion of GO. The *σ_prot_* decreased by the presence of 0.25% of GO but increased from 1.87 to 4.28 ×10^−4^ S·cm^−1^ when the proportion of GO increased from 0.25% to 0.75%. Finally, for 1.00% of GO it slightly decreased again. Given these results, the PVA-based crosslinked nanofibers combined with GO allowed better control of the proton conductivity. In general, it can be considered that they showed relatively high proton conductivity values, regardless of the small thickness of the nanofibrous membranes [10,19,64].

### 3.7. Hydrothermal Stability in Simulated Service Conditions

**Figure 9:**
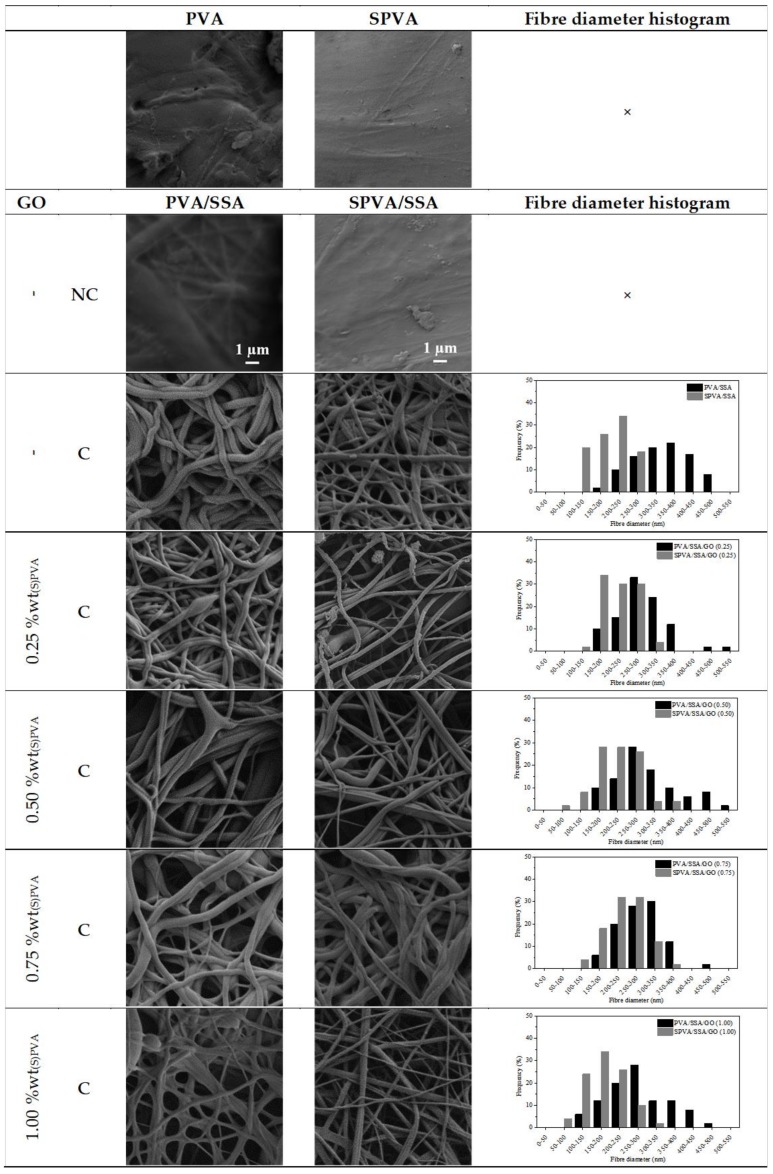
Surface morphology and fiber diameter histograms of the nanofibers of PVA and sulfonated PVA and their corresponding GO-based composites prior to and after immersion at 60 °C for 120 h. (C stands for crosslinked and NC for non-crosslinked; 10,000×.)

The evaluation of the behavior when exposed to service conditions is essential for the corroboration of the suitability of a polymeric material for a given application [65,66,67,68]. Polyelectrolytes may be used under aqueous or humid environments and should retain their nanofibrous morphology during application [54]. For this reason, the hydrothermal stability of the nanofibers was evaluated through immersion in ultra-pure water at 60 °C for 120 h, as a comparative accelerated procedure. The stability was assessed in terms of the change of the nanoscaled morphology and the average diameter of the nanofibers. The surface micrographs, along with the histograms of the diameters, are shown in Figure 9. Likewise, the average diameter of the nanofibers prior to and after immersion are gathered in Table 4.

Both PVA and SPVA non-crosslinked nanofibers completely lost the nanofibrous morphology after immersion. The nanofibers swelled until coalescence, which would result in the disappearance of the nanoscaled structure. In contrast, the crosslinked nanofibers retained their fibrous morphology after the hydrothermal treatment in all cases. A general increase in the average fiber diameter was observed, caused by swelling due to water absorption. The largest increase in the fiber diameter was perceived for the PVA/SSA and SPVA/SSA nanofibers. However, the use of SPVA promoted a lower increase after immersion, which may be correlated to the lower availability of hydrophilic sites, mainly hydroxyl groups, for the incorporation of water into the structure [54]. The presence of GO in the nanofibers resulted in a more compact structure, with less swelling capacity. In addition, the added GO could have established hydrogen bonds with the PVA molecules, as well as with the remnant water molecules, prior to immersion.

## 4. Conclusions

Functionalized nanofibrous membranes based in poly(vinyl alcohol) (PVA) or sulfonated poly(vinyl alcohol) (SPVA) combined with graphene oxide (GO) were developed by means of electrospinning. The subsequent crosslinking reaction with sulfosuccinic acid (SSA) brought thermal and hydrothermal stability, as well as the required electrical insulator performance and proton conductivity, to the nanofibers, especially relevant for polyelectrolyte applications. A good interaction of the GO nanoparticles with the PVA/SSA and SPVA/SSA matrices was found. The GO contributed strongly to retain humidity, both as free and bounded water, essential to promote proton conductivity. The crosslinked nanofibrous membranes were stable during simulated service conditions. Although fiber diameter increased after immersion, the presence of GO and the use of SPVA contributed to higher diameter stability. Finally, the developed nanofibrous composite membranes can be considered as good candidates for being exploited as components for the preparation of ionic multilayered polyelectrolyte membranes.

## Figures and Tables

**Table 1:** Calorimetric temperatures of the release of free (*T_free DSC_*) and bound (*T_bound DSC_*) water. Standard deviation between 1 and 2% omitted for the sake of clarity.

	Water Release	
	*T_free DSC_* (°C)	*T_bound DSC_* (°C)
PVA	70.7	-
PVA/SSA-NC	87.0	176.1
PVA/SSA	70.8	162.8
PVA/SSA/GO 0.25	70.5	169.4
PVA/SSA/GO 0.50	69.9	171.1
PVA/SSA/GO 0.75	63.3	170.9
PVA/SSA/GO 1.00	62.8	170.4
SPVA	70.6	179.2
SPVA/SSA-NC	89.4	169.5
SPVA/SSA	62.9	166.1
SPVA/SSA/GO 0.25	65.2	167.2
SPVA/SSA/GO 0.50	71.2	167.7
SPVA/SSA/GO 0.75	71.5	169.7
SPVA/SSA/GO 1.00	72.2	170.0

**Table 2:** Temperatures of the release of free (*T_free TGA_*) and bound (*T_bound TGA_*) water and the thermo-oxidative decomposition peak of the ‒OH (*T_d‒OH_*) and ‒SO_3_H (*T_d‒SO3H_*) groups along with the mass loss associated with each stage. Standard deviation between 1 and 2% omitted for the sake of clarity.

	Water Release			Decomposition		
	*T_free TGA_*	*T_bound TGA_*	Mass-Loss	*T_d‒OH_*	*T_d‒SO3H_*	Mass-Loss
	(°C)	(°C)	(%)	(°C)	(°C)	(%)
PVA	-	123.3	5.1	280.1	-	60.9
PVA/SSA-NC	56.5	172.0	32.9	271.1	313.1	60.9
PVA/SSA	54.5	158.6	22.9	271.4	320.2	51.3
PVA/SSA/GO 0.25	54.5	159.3	23.9	271.1	320.7	50.2
PVA/SSA/GO 0.50	56.4	174.9	26.3	268.2	320.9	51.8
PVA/SSA/GO 0.75	53.3	190.3	26.2	-	320.4	57.6
PVA/SSA/GO 1.00	55.3	197.8	24.2	-	322.8	55.8
SPVA	-	177.8	24.7	-	300.4	25.3
SPVA/SSA-NC	65.6	172.4	34.6	-	312.9	60.7
SPVA/SSA	51.3	167.6	30.2	-	315.2	60.2
SPVA/SSA/GO 0.25	56.3	162.9	27.7	-	320.4	60.1
SPVA/SSA/GO 0.50	54.7	161.5	26.9	-	320.5	61.1
SPVA/SSA/GO 0.75	55.3	169.7	27.8	-	321.5	59.2
SPVA/SSA/GO 1.00	53.1	171.2	26.5	-	320.2	60.4

**Table 3:** Proton conductivity (*σ_prot_*) of the nanofibers of PVA and sulfonated PVA and their corresponding GO-based composites.

	*σ_prot_* (S·cm^−1^ × 10^−4^)
PVA/SSA	2.5 ± 0.1
PVA/SSA/GO 0.25	2.7 ± 0.2
PVA/SSA/GO 0.50	3.8 ± 0.3
PVA/SSA/GO 0.75	3.9 ± 0.2
PVA/SSA/GO 1.00	4.4 ± 0.2
SPVA/SSA	3.3 ± 0.3
SPVA/SSA/GO 0.25	1.9 ± 0.2
SPVA/SSA/GO 0.50	2.3 ± 0.1
SPVA/SSA/GO 0.75	4.3 ± 0.2
SPVA/SSA/GO 1.00	3.8 ± 0.1

**Table 4:** Average diameter of the nanofibers of PVA and sulfonated PVA and their corresponding GO-based composites prior to and after immersion at 60 °C for 120 h. (NC stands for non-crosslinked.)

	Average Fiber Diameter (nm)		Variation (%)
	Initial	Final	
PVA	354 ± 120	×	×
PVA/SSA-NC	207 ± 60	×	×
PVA/SSA	198 ± 59	347 ± 69	+75.3
PVA/SSA/GO 0.25	229 ± 78	274 ± 53	+19.6
PVA/SSA/GO 0.50	241 ± 74	275 ± 60	+14.1
PVA/SSA/GO 0.75	249 ± 66	288 ± 58	+15.7
PVA/SSA/GO 1.00	238 ± 85	255 ± 86	+7.1
SPVA	200 ± 66	×	×
SPVA/SSA-NC	189 ± 48	×	×
SPVA/SSA	182 ± 33	233 ± 88	+28.0
SPVA/SSA/GO 0.25	186 ± 64	220 ± 49	+18.3
SPVA/SSA/GO 0.50	212 ± 42	219 ± 55	+3.3
SPVA/SSA/GO 0.75	201 ± 50	217 ± 61	+8.0
SPVA/SSA/GO 1.00	183 ± 58	199 ± 52	+8.7
